# Function Oriented Molecular Design: Dendrimers as Novel Antimicrobials

**DOI:** 10.3390/molecules22101581

**Published:** 2017-09-21

**Authors:** Sandra García-Gallego, Gianluigi Franci, Annarita Falanga, Rafael Gómez, Veronica Folliero, Stefania Galdiero, Francisco Javier de la Mata, Massimiliano Galdiero

**Affiliations:** 1 Organic and Inorganic Chemistry Department, Universidad de Alcalá, Campus Universitario, E-28871 Alcalá de Henares, Spain; sagg@kth.se (S.G.-G.); rafael.gomez@uah.es (R.G.); 2 Research Center on Bioengineering, Biomaterials and Nanomedicine (CIBER-BBN), 28029 Madrid, Spain; 3 Department of Experimental Medicine, II University of Naples, Via Costantinopoli 16, 80138 Naples, Italy; gianluigi.franci@unicampania.it (G.F.); veronica.folliero@unicampania.it (V.F.); 4 Interuniversity Research Centre on Bioactive Peptides (CiRPEB), University of Naples “Federico II”, Via Mezzocannone 16, 80134 Naples, Italy; stefania.galdiero@unina.it; 5 Department of Pharmacy, University of Naples “Federico II”, Via Mezzocannone 16, 80134 Napoli, Italy; annarita.falanga@unina.it; 6 John Felice Rome Center, Loyola University Chicago, Via Massimi 114, 00136 Roma, Italy

**Keywords:** dendrimers, structure, preparation, delivery, antimicrobial, antibacterial, antiviral, antiparasitic

## Abstract

In recent years innovative nanostructures are attracting increasing interest and, among them, dendrimers have shown several fields of application. Dendrimers can be designed and modified in plentiful ways giving rise to hundreds of different molecules with specific characteristics and functionalities. Biomedicine is probably the field where these molecules find extraordinary applicability, and this is probably due to their multi-valency and to the fact that several other chemicals can be coupled to them to obtain desired compounds. In this review we will describe the different production strategies and the tools and technologies for the study of their characteristics. Finally, we provide a panoramic overview of their applications to meet biomedical needs, especially their use as novel antimicrobials.

## 1. Introduction

Dendrimers [1,2] are a class of molecules that were first discovered before the 1970s and 1980s by research teams led by scientists such as Vögtle [3], Denkewalter [4], Tomalia [5] and Newkome [6]. Their name derives from the Greek word “dendron”, which means tree, for their characteristic branch-like schematic appearance. They are hyper-branched polymeric molecules with an almost perfect geometrical three-dimensional architecture and their exclusive branched topologies endow them functionalities that are completely different from those of linear polymers. The word dendrimer first appeared in 1984 in an article at the 1st International Polymer Conference (Society of Polymer Science, Kyoto, Japan), describing in detail the preparation of poly(amidoamine) (PAMAM) dendrimers.

Vögtle used cascade synthesis while Tomalia developed the divergent methodology. The divergent methodology based on acrylate monomers overcame the problems of low yields and purification which characterized the “cascade” synthesis and allowed the preparation in high yields of the first family of PAMAM dendrimers. These constructs had molecular weights ranging from several hundreds to over 1 million Daltons (i.e., generations 1–13) and were extremely successful, nowadays representing one of the most common methodologies used to make the market leader Starburst^®^ dendrimer family. The Vögtle cascade synthesis was initially troubled by scarce yields and difficulties in product isolation and purification which rendered its use unproductive to produce molecules large enough to exhibit the desired properties of dendrimers. It took a further ten years to allow the development of a thoroughly improved modification of the Vögtle method which was described by Wörner/Mülhaupt [7] (Freiburg University) and de Brabander-van den Berg/Meijer [8] (DSM group). The improved methodology rendered much easier the preparation of poly(propyleneimine) dendrimers and is still the most used commercial methodology to realize aliphatic amine dendrimers.

Dendrimers grow from the core into a globular structure and their sizes range between 2 and 5 nm, being among the smallest nanosystems developed so far. They essentially are made of different architectural domains: (1) the multivalent exterior with a considerable number of reactive sites; (2) the interior shells called dendrons; and (3) the core that serves for the attachment of the dendrons. Starting from the inner part of dendron toward the external, each ramification represents a generation (G-1, G-2, G-3), therefore, dendrimers of higher generations are larger, more branched and expose increased numbers of end groups at their surface compared to dendrimers of lower generations.

Different chemical procedures can be exploited for the construction of dendrimers, which will lead to specific characteristics of solubility, degradability and biological activity. The most common dendrimers used for biological applications are based on polyamidoamines (PAMAM) [9], polyamines [8], polyamides(polypeptides) [10], poly(aryl ethers) [11], polyesters [12,13], carbohydrates [14] and DNA [15,16]. By far the prevailing dendrimer scaffold is represented by the PAMAM dendrimers, which are purchasable on the market with an ample assortment of generations and surface functionalities.

There is a direct relationship between the dendrimer generations and the packaging of their peripheral groups, which promote the presentation of concentrated cargos of drugs or labeled molecules for both therapeutic and imaging applications. The coupling of drugs and targeting moieties are also based on the characteristic of terminal functionalities. Those structures represent a perfect launch pad for coupling several copies of a drug and/or ligand in general to the distal part of the dendrimers. The arising interactions happening between a dendrimer showing a multiple arrangement of ligands on its surface and the target containing several receptors lead to a tremendous increase in the avidity between the dendrimer and the cell [17,18]. Therefore, having several ligands (even if faint) coupled on a dendrimer scaffold can modify the structure into a high-affinity molecule.

Due to their peculiar characteristics, dendrimers have stimulated wide interest and present a wide range of applications in chemistry and biology, especially in drug delivery, imaging, antimicrobial applications and gene therapy.

Recently, several companies were created with a specific focus on the production of dendrimers for research applications, or for the development of dendrimer-based products. Tomalia started Dendritech (Mount Pleasant, MI, USA) in 1992, a company specialized in the production of PAMAM dendrimers. Dendritic Nanotechnologies Inc. (Mount Pleasant, MI, USA) was founded in 2001 and produced the Priostar™ dendrimer portfolio alongside the Starburst™ dendrimers. Another noteworthy company is Starpharma Pty. Ltd. (Abbotsford, Australia) that started its production in 1996 in Melbourne, Australia. Starpharma’s product of major interest is the microbicidal gel “VivaGel”, currently in phase 3 clinical trials for bacterial vaginosis with vaginal administration every other day for 16 weeks (~600 participants); two other phase 3 clinical trials for bacterial vaginosis with vaginal administration, once daily for 7 days (~500 participants total) are ongoing. VivaGel has so far proven useful in both treatment and prevention strategies. Nowadays, VivaGel is under investigation for prevention of genital herpes simplex virus type 2 (HSV-2), human immunodeficiency virus (HIV), and human papillomavirus (HPV).

In this scenario, other emerging companies, such as Polymer Factory (Stockholm, Sweden) and Colcom (Clapiers, France), both commercializing dendritic platforms for research activities, were funded. Polymer Factory produces dendrimers based on a 2,2-bis(methylol) propionic acid scaffold, while Colcom is specialized in distribution of poly-l-lysine (PLL)-based dendrimers. Finally, Tomalia has created a new company called NanoSynthons (Mount Pleasant, MI, USA), which is committed to the production of high-level nanoscale materials before distribution.

## 2. Structure of Dendrimers and Methods for Dendrimers Preparation

### 2.1. Synthesis of Dendrimers

The strategies for the preparation of dendrimers, known as “iterative methods of synthesis”, have evolved over the time [19,20]. Two different approaches—“divergent” or “convergent”—were initially used for the production of dendritic structures. In the first, the dendrimer growth is performed from the inner part to the periphery, building it up generation by generation. The main drawback of this approach is the huge flowchart of reactions on a single molecule that require a precise transformation in order to avoid defects. Through the alternative convergent approach [21] developed by Hawker and Frechét [22], the dendrimer growth starts from the external part and ends at the core, where the segments (dendrons) are linked together. In the convergent approach, with specific purification after each step, dendrimers without possible defects can be obtained. This approach permits the formulation of asymmetric dendrimer structures, for example by joining two different dendronic segments together in a controlled manner [23].

These double traditional routes are based on a repetition of activation and growth passages, which makes the synthesis of dendrimers laborious and time-consuming. New strategies have been introduced to facilitate this process, which are named “accelerated strategies” [24]. Different parameters have been exploited to accelerate the dendrimers synthesis: (i) building-blocks selection; (ii) reactions number; (iii) one-pot chemistries. Altogether these alternatives approaches have led to new strategies such as the “hypermonomer strategy”, the “double stage convergent growth” and the “double exponential growth” [24].

However, to further expedite the synthetic process, it is of chief importance to avoid the iterative activation steps. This objective can be achieved by the introduction of two or more robust and chemoselective reactions that can be performed during the orthogonal growth of dendrimers [24]. For example, the use of click-chemistry [25] is highlighted, including reactions such as the Diels-Alder, the Huisgen cycloaddition azide-alkyne terminal using a catalyst of copper at room temperature (CuAAC) and thiol-ene reaction. All of them are characterized by being simple, quantitative, with easy initiation and compatible with many solvents and functional groups.

### 2.2. Poly(propylene imine) Dendrimers (PPI)

**Scheme 1:**
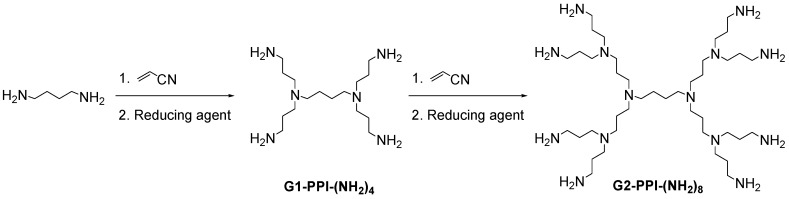
Synthesis of PPI dendrimers.

In 1978, Vögtle et al. [3] described the first “cascade synthesis” of a dendritic molecule, a divergent approach that lead to a poly(propylene imine) (PPI) skeleton (Scheme 1). It was based on a repetitive reaction sequence of Michael addition of a primary mono- or oligodiamine to acrylonitrile and subsequent reduction of the nitrile groups to generate primary amines. Several reducing agents [26] have been used for this purpose as Ni-Raney, LiAlH4, Co(II)-borohydride complexes, BH_3_·THF, etc.

Large scale synthesis of PPI was developed several years later, through a modification of Vögtle protocol, simultaneously by Mülhaupt et al. [27] and Meijer et al. [28]. The result is a highly symmetrical macromolecule before the spherical phase, due to the three-dimensional propagation. Their growth is limited by the drastic steric hindrance, as each generation doubles the amino substituents on the surface of the nanosystem. Consequently, the reaction stopped at a certain generation, being the 5th the highest generation synthesized of a PPI dendrimer. Large amounts of pure PPI dendrimers are currently commercially available from Aldrich Chemical Co. (St. Louis, MO, USA) and DSM (Heerlen, The Netherlands).

### 2.3. Carbosilane Dendrimers (CBS)

**Scheme 2:**
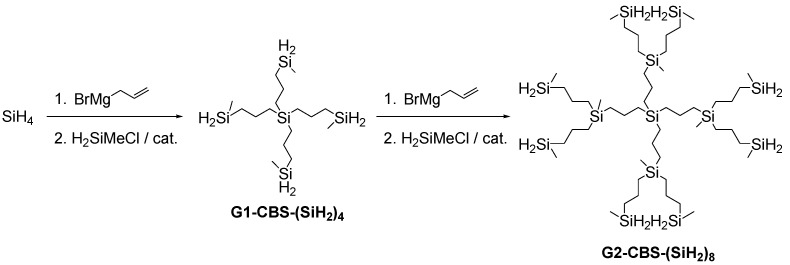
Example of a typical synthesis of CBS dendrimers.

Parallel to PPI dendrimer synthesis, Fetters [29] inaugurated in 1978 the synthesis of CBS dendrimers, the most important class of silicon-based dendrimers. A divergent strategy was used, through consecutive reactions of hydrosilylation of alkenyl groups and subsequent ω-alkenylations with Grignard reagents (Scheme 2). This synthetic route is highly versatile without drastic changes in the reaction conditions, being able to introduce different hidrosilylation reagents (HSiCl_3_, HMeSiCl_2_), ω-alkenylation reagents (allylmagnesium bromide, vinylmagnesium bromide) and also the core molecule (SiCl_4_, tetraallylsilane, tetravinylsilane, triallylmethylsilane). These changes clearly influence the dendritic structure, as can be observed in the different works by Made, Muzafarov, Zhou and Morán [30,31,32,33,34].

Several reactions, such as hydrosililation, click-chemistry or others, allow the subsequent functionalization of these carbosilane systems to water-soluble anionic and cationic macromolecules, with demonstrated applications as antiviral and antibacterial agents, or drug delivery molecules [35,36].

### 2.4. Peptide Dendrimers

**Scheme 3:**
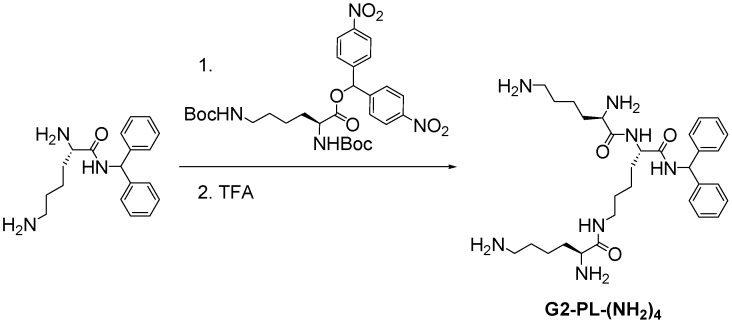
Synthesis of poly(l-lysine) dendrons proposed by Denkewalter [4].

At the beginning of the 1980s, Denkewalter [4,37] developed the first synthetic strategy leading to a well-defined poly(l-lysine) family of dendrimers [38]. Built from a benzhydrylamine core, the polypeptide dendrimer grew up to the tenth generation through a repetitive sequence of protection/deprotection using *N*,*N*’-bis(*tert*-butoxycarbonyl)-l-lysinenitrophenyl ester as reagent (Scheme 3).

For peptide-based dendrimers, the synthetic methodology is the usual for peptide compounds, with solid-phase approaches and combining divergent and convergent strategies, and also orthogonal methods for protection and chemoselective coupling allowing the hetero-functionalization of the dendritic core. This strategy also allowed the synthesis of the family of dendrons for the multiple antigenic peptide system [39] by Tam in 1988.

### 2.5. Newkome-Type Dendrimers or Arborols

**Scheme 4:**
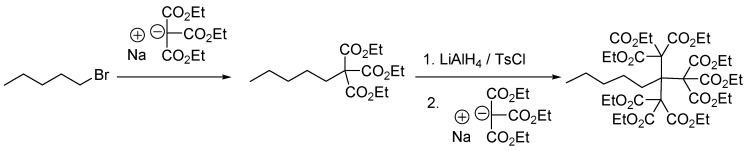
Synthesis of a typical Newkome-type dendrimer or arborol.

Also in the mid-1980s, Newkome et al. developed the synthetic strategy leading to the arborol dendrimers [6], through the nucleophilic substitution of 1-bromopentane by sodium triethylmethanetricarboxylate and subsequent reduction of the ester groups to an activated triol as a tosylate group (Scheme 4). These monocascade spheres possess a three dimensional microenvironment having the outer surface covered with polar functional groups, which lead to a micelle-like system.

### 2.6. Polyamido(amine) (PAMAM) Dendrimers

**Scheme 5:**
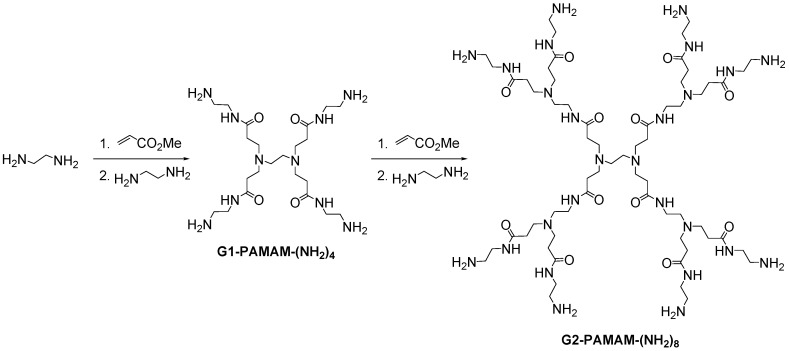
Synthesis of PAMAM dendrimers.

During the same decade of the 80s, Tomalia [5] published the first synthesis of PAMAM dendrimers, based on ethylenediamine core. The divergent strategy is based in a two-step sequence of a Michael-type addition of methyl acrylate to amines and subsequent attack with ethylenediamine to form amide bonds (Scheme 5).

PAMAM dendrimers are probably the most studied dendritic architectures, reaching molecules up to the 10th generation. Moreover, a large variety of PAMAM systems with different cores and terminal groups are commercially available.

### 2.7. Phosphorus Dendrimers 

**Scheme 6:**
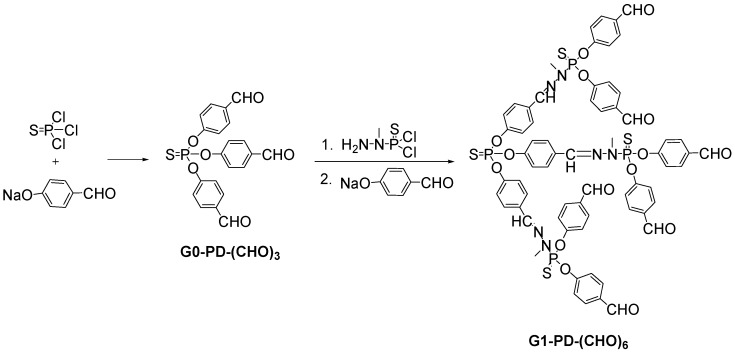
Example of synthesis of phosphorus-containing dendrimers (PD).

In the early 90s, Rengan and Engel [40] initiated the synthesis of phosphorous-based dendrimers, prepared through the splitting of the ether bonds by trimethylsilyl iodide and subsequent attack of a phosphine with three ether groups. Later on, the first neutral phosphorus-containing dendrimer was described by Majoral et al. [41,42,43,44] prepared through the reaction of a core containing “*n*” equivalents of *p*-hydroxybenzaldehyde and subsequently condensation of the aldehyde group with H_2_N-N(Me)-P(S)Cl_2_ (Scheme 6). Afterwards, they have given continuity to these studies by generating a great diversity of families, some of them up to the 12th generation. Different dendritic architectures belong to this group, where the phosphorus atom is bonded to other heteroatoms such as N and O, and also is located in the core, the branching point or/and the periphery. Several systems are also functionalized with either positive or negative charge, in order to acquire water-soluble properties for biological applications.

### 2.8. Other Types of Dendrimers 

**Figure 1:**
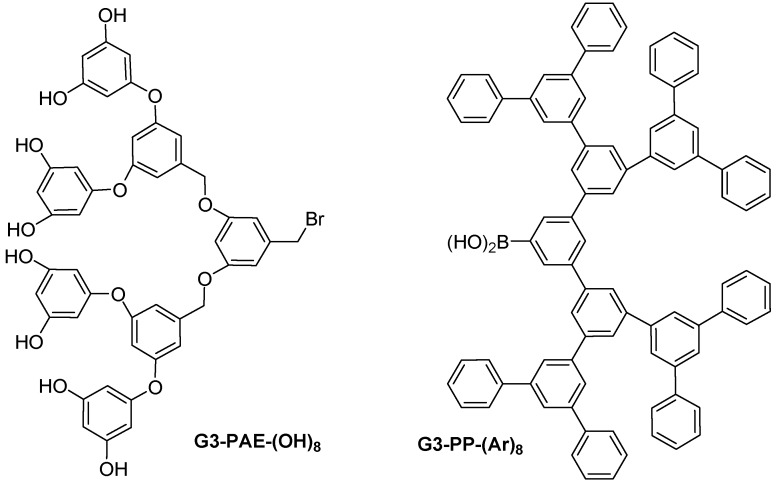
Example of 3rd generation poly(aryl ether) and poly(phenylene) dendrimers.

Also in the 90s, converging routes [21] began to appear with the poly(aryl ether) dendrimers developed by Hawker and Fréchet [11,22] and the poly(phenylene) (PP) dendrimers reported by Miller and Neenan [45] (Figure 1). Poly(aryl ether) (PAE) dendrimers were prepared by using 3,5-dihydroxy-benzyl alcohol as the monomer. The two phenolic groups of this monomer were coupled to benzylic bromide (bromination step), producing the two new ether linkages of the second generation benzylic alcohol. The focal benzylic alcohol functionality was then activated for the following reaction (coupling step) with carbon tetrabromide and triphenylphosphine affording brominated dendron. Subsequent repetitions of the Williamson coupling and bromination steps allowed the production of the different generations of dendrimers. Poly(phenylene) dendrimers involved the Suzuki coupling of arylboronic acids with monomer 3,5-dibromo-1-(trimethylsilyl) benzene. Conversion of the trimethylsilyl (TMS) protecting group of the products to the boronic acid functionality enabled further coupling to the monomer. The rigid repeat units of these molecules lead to dendritic structures with defined shapes and diameters. Other noteworthy synthesis include those of poly(phenylene), poly(alkyl ester), poly(aryl alkene) and poly(alkyl ether) dendrimers [21].

These convergent approaches have subsequently given rise to a great family called Janus-type dendrimers [23], with multiple topologies. These macromolecules are constituted of two dendrimeric wedges and terminated by two different functionalities. Janus dendrimers have been prepared from several types of structures (benzyl ether, phenylene, phosphorus, PAMAM, DAB, lysine, alkyl ether, ester, amide, etc.).

**Scheme 7:**
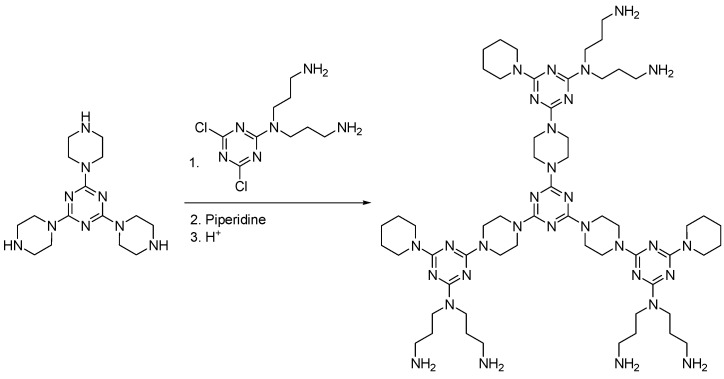
A divergent route adopted for kilogram-scale synthesis of triazine dendrimers [46].

Perhaps among the most recent dendrimers are triazine dendrimers developed by Simanek [46]. Their synthesis is based on the sequential replacement of trichlorotriazine with nucleophilic amines, obtaining dendrimers with high purity and yield, both by a divergent and convergent strategy [47] (Scheme 7).

## 3. Characterization of Dendrimers

### 3.1. Spectroscopic and Spectrometric Methods

#### 3.1.1. Mass Spectrometry (MS)

MS is an analytical technique that allows the determination of the molecular weight, the structural defects in dendrimers, the polydispersity or the purity. It also allows the confirmation of the monomer assembly in the branches, from the fragmentation pattern. Several techniques have been used, such as electron impact (ESI-MS), fast atom bombardment and matrix-assisted laser desorption ionization (MALDI) mass spectroscopy [48], although the results need to be interpreted with care [49]. MALDI-TOF mass spectroscopy technology represents the best option in order to evaluate the molecular weight of the major-generation dendrimers in combination with the Gel Permeation Chromatography GPC analysis and diffusion-ordered NMR spectroscopy (DOSY NMR) are currently explored [50]. Nowadays, accumulating evidence shows the huge potential of mass spectrometry for the analysis of non-covalent dendritic host-guest complexes [51].

#### 3.1.2. Nuclear Magnetic Resonance (NMR) Spectroscopy

NMR spectroscopy is a paramount technology for dendrimer characterization. In details this approach permits the dynamics structure determination in solution. Both mono (^1^H-, ^13^C-NMR) and bidimensional (2D-NOESY, 2D-TOCSY, DOSY, etc.) experiments are widely used to characterize the synthesis of dendrimers, molecule conjugations, conformational changes, group mobility, etc. For organic dendrimers, such as PPI or polyphenylester dendrimers, ^1^H and ^13^C are the most used [28,52], while for heteroatom-containing dendrimers the resonance of the heteroatom can afford very valuable information, as it happens with CBS dendrimers [36,53] (^29^Si-NMR) or phosphorus dendrimers [54] (^31^P-NMR). ^15^N-NMR has been also used to detect their selective protonation first on the surface of the second generation, then at the core, then at the level of the first generation [55].

Chemical shift titration experiments give information on the interaction properties between dendrimer and guests [56], and can be used for the calculation of binding parameters and the spatial conformations within the dendrimer/guest complexes.

#### 3.1.3. Electron Paramagnetic Resonance (EPR)

EPR has been verified as a very powerful tool that provides unique information for the characterization of dendrimer structures [57]. By using spin probes, it can widely and precisely inform about dendrimer structure and also their interaction with other molecules such as proteins and drugs. For instance, using Cu(II) as a spin probe, anionic decorated dendrimers with carbosilane [58] and PPI [59] skeleton were analysed, finding a different behaviour towards metal coordination, which is related to their dendritic structure. On the other hand, computer-aided EPR analysis by means of the selected spin probe 4-octyl-dimethylammonium, 2,2,6,6-tetramethylpiperidine-1-oxyl bromide (CAT8) demonstrated the kinetics of amyloid and prion fibril formation in the absence and presence of dense shell sugar-decorated dendrimers [60].

#### 3.1.4. Fluorescence Spectroscopy

Valuable information regarding size and shape of dendrimers, and their interaction with drugs can be obtained by using fluorescence spectroscopy. It was used to characterize pyrene-containing dendrimers [61] as fluorescence materials, with future applications as biological probes, optoelectronic devices or in photodynamic therapy, as well as peptide dendrimers consisting of fluorescent groups surrounding by branched amino acids [62]. Paulo et al. [63] reported a fluorescence decay on a phthalocyanine-PAMAM dendrimer complex, attributed to electron transfer between excited-state phthalocyanine and the dendrimer’s tertiary amines, which are deprotonated at higher pH and therefore can act as efficient electron donors. Cationic CBS dendrimers containing eugenol linkers developed by Rasines et al. [64] are able to interact with anionic drugs and proteins, as it has been evidenced by the decrease in fluorescence and other NMR experiments. This technique also demonstrated the pH-dependent fluorescence emission of some dendrimers, like OH-, NH_2_- and carboxylateterminated PAMAM and NH_2_-terminated PPI or ammonium decorated CBS dendrimers [35].

#### 3.1.5. Infrared and Raman Spectroscopy

Infrared spectroscopy is mainly applied to routine analysis of the changes in the dendrimer synthetic process, for instance to observe the degree of transformation of nitrile groups to amino groups in the synthesis of PPI dendrimers [28]. Other information, such as the characterization of delocalized π-π stacking interactions between end groups of modified PAMAM dendrimers, could be also analysed by near IR spectroscopy [65].

Moreover, FTIR and FT-Raman spectroscopy provides exclusive precise information on the structure of nanoscale materials, which were impossible to obtain with previous technologies. Information on phosphorus-containing 12 generation dendrimers spectra [66] highlight that, for generations higher than 6, the tight conformations of the terminal groups are disturbed by the steric congestion and the rather strait-laced repeated units with small conformational flexibility are the baseline of phosphorus-containing dendrimers up to 11 generation microstructure.

#### 3.1.6. UV-Vis Spectroscopy

This technique has been widely applied to monitor dendrimers production as the intensity of the absorption band is directly proportional to the number of chromophoric units. It can test the homogeneity of azobenzene decorated PPI dendrimers [67] or double-layered CBS dendrimers [68], although deviations from the Beer-Lambert law could be detected in some cases, probably due to some other factors involved in the absorption of the dendrimers, like the rigidity of the system and the influence of the peripheral groups as observed in cationic CBS dendrimers [35].

Other morphological information could be assessed through UV-Vis, as demonstrated by Hawker et al. with dendritic polyethers with a solvatochromic probe in their core [69], observing an impressive modification in the absorption maximum from G3 to G4 representative of a transition from an open to a more spherical shape. 

### 3.2. Chromatographic Techniques

#### 3.2.1. Gel Permeation Chromatography (GPC)

This technique is commonly used to find information on the composition of dendrimers, including their polydispersities. Novel polyesteramine dendrimers developed by Akiyama et al. [70] by divergent and convergent methods were found to have narrow polydispersities by GPC analysis, ranging values of 1.01–1.07. Characterization of CBS dendrimers derived from 1,3,5-trihydroxybenzene [36] with different substituents in the periphery shown different polydispersities depending on the external group, revealing values of 1.05–1.18 for allyl or ester decorated systems and slightly higher for amine dendrimers (1.13–1.40). In all cases, these values confirm the high monodispersity degree of these systems.

For GPC analysis of dendrimers, they are usually used polystyrene standards due to the difficulties of obtaining other standards of known relative molar mass and polydispersity [71]. However, several studies are demonstrating that universal calibration curves built according to the data of CBS dendrimers 3 × 3, 4 × 3 practically coincide with the one for polystyrene standards, approving application of universal calibration for determine molecular masses and sizes of a wide range of dendrimers and hyperbranched polymers. 

#### 3.2.2. High-Performance Liquid Chromatography (HPLC)

Although HPLC is limited to relatively low molar mass compounds, it gives useful information on the homogeneity of dendrimers and dendrons, and also their impurities. Combined with other techniques, such as potentiometric titration and mass spectrometry, HPLC provided a quantitative evaluation of G5 PAMAM dendrimer defects [72], identifying, isolating and characterizing the major structural defects of this system. Reversed-phase HPLC allowed the individual component separation of dendritic macromolecules substituted with various trimethylsilyl and dodecyl groups [73], while size-exclusion chromatography only provided a rough picture of the composition of the mixture.

### 3.3. Scattering Techniques

Scattering techniques [74], such as SANS, QENS, SAXS, and DLS have been shown of paramount importance for the investigation of dendrimer interfacial behaviors at the nanoscale level. Moreover, these approaches represent a cardinal point in the definition of structure, conformation, shape, and dynamics of dendrimers. 

#### 3.3.1. Small Angle Neutron Scattering (SANS)

The SANS methodology reveal accurate information on the interior part of the entire dendrimer. It has been used to calculate the molecular weight of PPI [75] and PAMAM [76] dendrimers, and also the location of the end groups by labeled (deuterating) them. This study revealed a different location in both dendrimers, the end groups being concentrated near the periphery in PPI dendrimers [77] whereas throughout the structure in PAMAM systems [78].

#### 3.3.2. Quasi-Elastic Neutron Scattering (QENS)

The G5 PAMAMs in D_2_O solution as a function of protonation from a dynamical perspective was investigated by Chen et al. [79]. They used a combined QENS and high-resolution solution NMR spectroscopy methodology. QENS accurately demonstrated the local motion of dendrimer segments, reporting an augmented local motion at increasing molecular charges, which is opposite to the hypothesis that increased protonation should stabilize the dendrimer and avoid the local motion.

#### 3.3.3. Small-Angle X-ray Scattering (SAXS) 

Although SAXS and SANS use different scattering elements (X-rays and neutrons, respectively), their data analysis are similar in many ways. SAXS was used to characterize the single-particle scattering elements formed by different species of dendrimers [80], and was also useful to examine the advancement of intramolecular organizations of PAMAMs, producing a maturation from “star” to “sphere” organizations [81].

#### 3.3.4. Dynamic Light Scattering (DLS)

DLS is abundantly employed to analyse the structure of macromolecules. For instance, Jachimska et al. [82] performed DLS experiments to measure the electrophoretic mobility and diffusion coefficients of G6 PAMAM dendrimers in an aqueous solution, determining the effective charge and the hydrodynamic radius these molecules. DLS, in combination with other techniques, was used to evaluate the interactions between cationic (G3, G4, and G5) and anionic (G4.5) (PAMAM) dendrimers [83], revealing that the formation of anionic-cationic dendrimer aggregates is an enthalpy-driven process. 

### 3.4. Microscopy

#### 3.4.1. Atomic Force Microscopy (AFM) 

AFM allows the characterization of the surface topography of dendrimers adsorbed onto a surface such as silica. AFM measurements of PAMAM dendrimers [84] have shown the strong impact of substrate and pH of deposition solution upon the height, diameter, and volume of these soft, deformable dendrimers.

This technique has also been used to characterize several dendrimer conjugates, in order to determine their precise size. This is the case of anti-HIV oligodeoxynucleotides (ODNs) carried by CBS dendrimers [85], or doxorubicin loaded poly(l-glutamic) acid dendrimers [86], both also characterized by Transmission Electron Microscopy (TEM). 

#### 3.4.2. Scanning Tunnelling Microscopy (STM)

While AFM permits a precise measurement of the height, STM allows the production of high-resolution images and a rigorous determination of the lateral dimension of single dendrimers. Shi et al. [87] developed a detailed protocol to visualize intramolecular structure of indomethacin-loaded G4 PAMAM-OH dendrimers. The combination of both microscopy techniques enables insightful and fundamental information in the context of molecular level location and load of drug molecules, as well as the stability of drug-carrier complex. 

### 3.5. Electrophoretic Techniques

Several dendrimers may carry multiple charges and can be studied by electrophoretic techniques [88]. Pulse Acrylamide Gel Electrophoresis (PAGE) [89] and capillary electrophoresis (CE) [90] provide useful information about e.g., purity, homogeneity or electrophoretic mobility.

Succinamic acid surface-modified PAMAM dendrimers synthesized by Shi et al. [89] were analysed by different electrophoretic techniques. Native (gradient) PAGE allowed the separation of different generations as a consequence of the gel filtration effect, therefore, assessing purity and homogeneity, while sodium dodecyl sulphate (SDS)-PAGE allowed the estimation of their molecular weight based on a protein standard. The developed capillary zone electrophoresis (CZE) method concluded that only the charge/mass ratio and the electro-osmotic flow influence the separation.

Dendrimers can be characterized by CE, but also can be used to improve the separation of diverse compounds through this technique. Montealegre et al. reported a better separation and higher resolution of vegetable proteins by using CBS anionic dendrimers as nano additives in CE [91].

The combination of capillary electrophoresis and mass spectrometry (CE/MS) provides the opportunity to detect closely related compounds and isomers. Stöckigt et al. [92] separated and identified polydisperse dendrimeric diaminobutane(DAB)-based polynitriles (DAB-dendr-(CN)8) and synthesis by-products by using the online coupling of capillary electrophoresis with a sector mass spectrometer via an electrospray ionization (ESI) source.

Electrophoretic techniques are also commonly used to characterize different dendrimer conjugates, such as dendriplexes. Complex formation and stability, charge and molar ratio and the DNA conformation in the dendriplex are possible to study using agarose gel electrophoretic method [93]. Weber et al. [94] characterised amino-terminated CBS dendrimers created to preserve and deliver small interfering RNA (siRNA) bound to dendrimer surface by electrostatic interactions. Stability of the complex was analysed performing heparin competition assays on agarose electrophoretic gel system and PAGE system. These studies demonstrated that the complex was highly resistant to degradation by RNase, therefore the CBS dendrimer can exert a protective influence on siRNA in the presence of RNase.

### 3.6. Other Types of Techniques

#### 3.6.1. X-ray Diffraction

Dendrimers are usually amorphous solids with lack of long-range order in the condensed phase. This is the reason why X-ray diffraction is generally a fruitless technique to precisely determine the chemical composition, size and shape of dendrimers. However, some authors have determined the structure of several dendrimers. Brewis et al. [95] characterized the X-ray structure of a silicon phthalocyanine with axial second generation dendritic substituents. Other techniques, such as small and wide-angle X-ray diffraction on powder and oriented fibers combined with electron density maps, endow precise information on the 2- or 3-D structure of dendronized polymers, as shown by Percec et al. [96,97].

#### 3.6.2. Acid-Base Titration

Dendrimer acid-base titration has been used to get information about their behavior at different pH values, especially from poly(propylene imine) (PPI) dendrimers and PAMAM dendrimers. In both cases, they present a skeleton where tertiary amines can be protonated, thus being pH dependent. The data is usually interpreted with a site binding model which involves the microscopic ionization constants for the different groups in the dendritic structure. The protonation of PAMAM dendrimers [98] first involves protonation of primary amine groups at the external rim of the dendrimer at high pH, while the tertiary amine groups in the dendrimer core protonate at lower pH, and the last group to protonate at low pH is a central tertiaryamine. For carboxylate and sulfonate decorated PPI dendrimers [59], the experimental results agreed with the theoretical calculations, and revealed that at physiological pH the predominant macrospecies of the first generation dendrimers present some nitrogens protonated, from the core and the first layer while all the anionic groups are deprotonated.

## 4. Applications of Dendrimers

Dendrimers have been successfully used in nanotechnology and in particular in medicinal chemistry. Their branched architecture appears to be essential for their application and the plethora of ligands on their external shell has proved to be of major impact for inhibiting the multivalent adhesion activities between viruses, bacteria and cells, proteins and combinations thereof. Dendrimers can act as drugs themselves or they can be exploited as carriers for several different drugs molecules. Dendrimers can be modified with multiple groups in order to allow diverse labels, targeting ligands and other molecular entities to be included within a unique delivery and single entity; moreover, the improvements achieved in dendrimer synthesis have also allowed the finely tuning of two or more constituents at different ratios on the same dendrimeric scaffold. The dendrimer drug interaction or drug loading may be achieved in several ways: (1) encapsulation in the interior of the dendrimer; (2) electrostatic encapsulation; (3) covalent conjugation. Furthermore, by a careful choice of their chemistry dendrimers can be rendered biocompatible for their controlled degradation in order to reduce any eventual environmental hazard. In the remaining of the review we will describe some of their biomedical applications for drug delivery, with a particular insight for their antimicrobial properties.

### 4.1. Drug Delivery and Cell Transfection

The development of an efficacious drug delivery tool is pivotal to enhance the pharmacological functioning of drug molecules. The attachment of a drug to a proper carrier allows the improvement of its aqueous solubility, the expansion of its circulation half-life, the targeting for specific tissues, the enhancement of its ability to cross biological barriers, which maximizes drug bioavailability to tissues of interest while attenuating drug exposure of healthy tissues, therefore, resulting in augmented therapeutic potency. As a matter of facts, dendrimers have emerged as an alternative to linear polymeric carriers for delivery of drugs also thanks to their multivalency which allows the coupling of drug molecules and/or ligands in a precisely organised fashion to boost the binding effect and the affinity of therapeutic molecules to receptors with a synergistic interaction. Furthermore, dendrimers are of great interest for drug delivery because of their ability to cross cell membranes, including the blood-brain barrier (BBB) and have been effectively used as carriers of many pharmaceuticals.

The antitumor drug cisplatin (20–25% by weight) has been coupled to the surface of a G4 carboxylate-PAMAM dendrimer, leading to a considerable increase (at least 10-fold) in cisplatin solubility. Coupling of cisplatin induced a cross-linking effect between dendrimers, resulting in the formation of higher order structures with diameters of 30–40 nm. It was reported that when cisplatin was coupled to dendrimers, its release was slower, therefore a higher accumulation in solid tumors and lower toxicity was granted [99].

PAMAM dendrimers have been applied to the delivery of methotrexate (MTX). Treatment of mice, bearing subcutaneous cervical carcinoma derived by KB tumor cells, that overexpress the folic acid receptor, with MTX-folate-fluorescein modified dendrimers resulted in a notably lowered amount of tumor expansion [100]. Dendrimers of small diameter (<5 nm) were rapidly cleared from blood through kidneys; such rapid elimination avoids the need that the utilized carrier has to be biodegradable to prevent bioaccumulation [100].

PAMAM dendrimers with carboxymethyl polyethylene glycol (PEG) and encapsulating the anticancer drug 5-fluorouracil showed acceptable drug loading, reduced release rate and hemolytic toxicity compared to the non-PEGylated dendrimer [101].

Ibuprofen encapsulated in PAMAM dendrimers was efficiently conveyed to lung epithelial carcinoma cells [102]. The bioavailability of indomethacin in transdermal delivery applications was enhanced when linked to PAMAM dendrimers [103]. PAMAM dendrimers complexed with puerarin have been used as ocular drug delivery vehicles and have shown to produce longer ocular persistence times in rabbits compared with puerarin eye drops [104].

A poly(amide)-based dendrimer was recently produced and functionalized with the membrane-interacting peptide gH625 derived from the herpes simplex virus type 1 (HSV-1) envelope glycoprotein H. The conjugation to the peptide was shown to increase significantly the penetration into the cellular matrix. It was shown that the peptide-functionalized dendrimer is rapidly taken into the cells mainly through a non-active translocation mechanism [105,106,107].

The BBB represents an impermeable barrier for the pharmacological treatment of several neurological disorders, such as neuro-inflammation, glioma, infections and neurodegenerative diseases. The BBB impedes, by a physical point of view, the diffusion of drugs and other molecules from the systemic circulation into the brain. Dendrimer-based drug delivery nanosystems have been shown to be able to adopt alternative routes of drug transport across the BBB. New evidence highlights the possibility of the involvement of endocytosis mechanisms mediated by clathrin and caveolin [108] and via specific receptor-mediated pathways driven by brain-targeting ligands conjugated to the dendrimer surface. Examples of delivery across the BBB include administration of drugs targeting Alzheimer’s disease [109].

Cationic dendrimers have been shown to penetrate in vitro epithelial monolayers mimicking the BBB; it has been proposed that they initially bind electrostatically the cell membrane and transiently reduce the trans-epithelial electrical resistance, indicating an opening of tight junctions [110]. Unfortunately, on the other hands, their activity in vivo is limited due to the fast clearance and residual toxicity occurrence.

PAMAM dendrimers have been used to enhance the delivery of the anti-inflammatory drugs, such as the *N*-acetylcysteine (NAC) to the central nervous system (CNS) of rabbits with laboratory-induced cerebral palsy. These dendrimers could be selectively targeted to inflammatory activated microglia and astrocytes within diseased brains [111].

When administered systemically, they are able to significantly improve the motor functionality in diseased rabbits, as opposed to the administration of 10-fold higher doses of free NAC. Moreover, there was no evidence of toxicity at the doses used. 

A dual targeting drug carrier composed of a fourth generation PAMAM dendrimer (G4) conjugated to transferrin on the exterior and tamoxifen in the interior has been produced for enhancing the BBB transportation and improving drug accumulation in glioma cells [112].

Among the applicative fields of dendrimers, gene therapy is one of the most effective technologies to treat major infectious diseases, cancer and genetic disorders. A safe and highly efficient gene carrier is necessary to transfer genes through the cell membrane into the nucleus. Currently, liposomes and genetically engineered viruses are used for this purpose; but more recently PAMAM dendrimers have been used for this aim in vitro and in vivo [113], thanks to their ability to form complexes with negatively charged genetic materials. Upon the advantages of dendrimers, the non-immunogenic nature of dendrimers is one of the major advantages. Moreover, dendrimers showed the highest efficiency of transfection compared to the other non-viral systems, without the side effects of viral systems. Several non-toxic and efficient multifunctional dendrimer-based conjugates for gene delivery have been reported, but to further exploit the technology, several functionalizations to improve efficiency of transfection and reducing cellular toxicity are available.

Cationic PAMAM dendrimers decorated with primary amines on their surface were initially used as gene delivery systems and compared to the traditional polyamine transfection agents showing higher efficiencies and lower toxicities with the generation of DNA complexes [114]. Based on this characteristic, kits based on dendrimers are commercialized under the logos of SuperFect Qiagen. Among their application as transfectant agent, for instance dendrimenrs were used for the treatment of subcutaneous tumors in a murine model. 

In analogy to other cationic carriers, the toxicity associated with the positive charge of the PAMAM dendrimers needs to be addressed in order to successfully use them in the clinic.

PAMAM dendrimers toxic reduction was achieved with surface coating with poly(ethylene glycol) and use of disulfide linkages. Moreover, this modification also increased efficiency of transfection [115,116,117]. The same results were obtained via conjugation of the dendrimers with membrane targeting peptides, alkyl chains, sugars and arginine/lysine compounds [118,119,120,121,122,123].

PAMAM G4 dendrimer’s surface decorated through an isothiocyanate linker with paromomycin, neamine and neomycin showed increased gene delivery activities compared to the original dendrimer, along with lower cytotoxicity [124]. Moreover, unlike PAMAM G4, the presence of serum did not affect the transfection efficiency, indicating them as an attractive tool for in vivo applications. 

PLL dendrimers with the *tris*(2-aminoethy)amine core have been produced and studied for their applications in gene transfection [125]. In vitro and in vivo cytotoxicity experiments highlighted the high biocompatibility of those dendrimers. Moreover, in vitro gene transfection efficiency of G5 was higher than other dendrimers with or without the presence of serum [125].

An increased siRNA gene silencing and doxorubicin co-delivery was obtained with the silsesquioxane cubic core based PLL dendrimer, which showed highly gene silencing in U87-Luc cells thanks to the combinatorial effects of siRNA and cytotoxicity activity [126].

In the same time, PLL dendrimers have lower gene transfection efficiency compared to other traditional cationic polymers such as PAMAM dendrimers, this implication reflected in a lower interest of applications.

PPI, carbosilane, phosphorus containing dendrimers, and amphiphilic dendrimers have been studied for their gene delivery potential, biodegradability, and serum stability [127].

Deeper characterization of DNA complex efficacies in gene delivery properties of a nitrogen-core poly(propyl ether imine) (PETIM) dendrimer [128] showed that the dendrimers enfolded the DNA without bending of it. Concerning the variability on cell line used, these dendrimers exhibited 50 to 100 times better transfection efficiency compared to poly(ethylene imine) polymers [128].

Recent studies showed the possibility to realize water soluble dendritic pillar[3]arene derivatives with 20 and 40 peripheral ammonium groups [129]. As described before for others dendrimers, these dendrimers forming stable nanoparticles with plasmid DNA provided encouraging results in terms of transfection efficiencies in vitro using HeLa cells with low toxicity.

Other recent dendrimers with a star-shaped polymer (PP-PLLD) formed by a porphyrin (PP) core and PLL dendron arms were synthetized and tested for the capability to transfect eukaryotic cell lines with a GFP reporter plasmid (pEGFP) [130]. PP-PLLD was able to form compact complexes with pEGFP. In vitro assays also indicated that PP-PLLD showed photo enhanced gene transfection efficiency [130].

A further innovative approach was the construction of cyclodextrin derivative containing poly(l-lysine) dendrons, which could form colloidal nanocomplexes with pDNA with gene transfection efficiency tested on MCF-7 cells (human breast cancer) [131]. Furthermore, these dendrimers showed a higher loading capacity and a sustained release behaviour for poorly water-soluble methotrexate with anticancer activity. Together these properties can be useful in a combined delivery of plasmid DNA and lipophilic anticancer drugs [132].

### 4.2. Antimicrobial Activity

The emergence of resistance towards antibiotics has forced scientists to look for novel kinds of molecules endowed by higher efficacy, selectivity, and safety. One of the most recent goals in this field concerns the use of nanomaterials as alternative antimicrobial agents thanks to their specific physical and chemical properties.

Numerous studies provide evidence that various classes of dendrimers are active against different viruses, bacteria, and fungi targeting or inhibiting virulence factors involved in microbial pathogenesis and thus may represent very attractive compounds to be developed as novel drugs. 

Dendrimers can exert their effect via different mechanisms of action, which are related to the multivalency of the nanomolecule and strongly affected by the nature and number of their functional surface groups. The ability to manipulate and change the type of these functional groups opens the possibility to design and synthesize specific dendrimers, which may be able to block receptors used by pathogens for adhesion, cell entry and dissemination.

#### 4.2.1. Antibacterial Activity

The potential of using dendrimers as antimicrobial agents has been recognised and analysed to a broad extent over the last 10–15 years. Dendrimers can play their effect according to different mechanisms of action, generally associated with the multivalency of the branched scaffold. Recent studies drew attention to the plasticity of dendrimer synthesis to produce antibacterial dendritic structures with specific dimensions and surface modifications. Often antibacterial dendrimers expose cationic surface functionalities such as amines or tetraalkyl ammonium groups in order to increase the attractive forces against the bacterial membrane that has a negatively charged surface and in fact they are effective against Gram-negative and Gram-positive bacteria [133,134]. In particular, the main mode of action of these cationic nanomolecules is dependent on the electrostatic interactions between positively charged dendrimers and the negatively charged bacterial membrane resulting in the increase of membrane permeability which is able to lead to bacterial lysis and cell content release. On the other hand, anionic group functionalization showed a lower activity and efficiency [135]. Ladd et al. compared the two first generation of cationic dendrimers, the amine terminated (G1-NH_3_^+^) and the free OH (G1-OH) for their bactericidal activity on *Escherichia coli*. Compared to the G1-NH_3_^+^, G1-OH did not show considerable bactericidal activity, highlighting the predominant role of cationic groups [136]. Despite promising biocidal activity, the cationic dendrimers were inherently toxic to mammalian cells. Several studies were conducted to preserve the antibacterial effect with a significant reduction in term of toxicity on eukaryotic cells. For example, the modification of dendrimers’ amino groups with PEG chains PAMAM showed a drastic reduction of cytotoxic effects without antimicrobial capacity compromise [137]. De Queiroz and colleagues conjugated dendritic polyglycerol (PGLD) with chitosan to realize PGLD–chitosan dendrimers able to suppress bacterial proliferation of *Staphylococcus aureus* and *Pseudomonas aeruginosa* [138]. Active antimicrobial compounds coupled on dendrimers have been shown to have increased antimicrobial activity. In fact, many dendrimers bearing as terminal groups antimicrobial compounds such as quaternary ammonium [139], boron complexes [138] and peptides [140,141], showed increased activity. Dendrimers can be designed to contain hydrophobic segments that make them work like the pore-forming-helical peptides (e.g., the defensins) and constitute highly membrane-active drugs [142]. PAMAM dendrimers induce pore formation leading to destruction of bacterial membrane [143]. To inhibit bacterial growth, polycationic dendrimers take advantage from the physical disruption and eventually destruction of the bacterial membrane rather than the interaction with specific proteins. Their positive charge favours the interaction with negatively charged bacterial envelopes while reducing the interaction with zwitterionic eukaryotic membranes. Increasing interest as bacterial targets is focus on peptide-dendrimers. Multiple dipeptides composed of tryptophan and arginine residues were coupled on dendrimers synthetized by Kallenbach et al. The chemical characteristic mediated by arginine residues, positively charged, confers the possibility to mediate electrostatic interactions with the cell wall. The tryptophan residues with the lipophilic parts are able to interact with membranes compromising their integrity [144,145]. In this scenario a very interesting dendrimer modified with peptides was obtained with the G3KL dendrimer against *P. aeruginosa* and *Acinetobacter baumannii*. The peptido-dendrimer structure contained dipeptides (lysine and leucine), coupled via lysine residues and proved to be active also against carbapenemase producer clinical strains [145,146]. Peptido-dendrimers based on di-, tetra-, or octavalent lysine cores bound with tetra-(RLYR) or octapeptides (RLYRKVYG) exhibited a strong antibacterial activity against both Gram-positives and Gram-negatives, but a major increase of the activity was observed against Gram-negative bacteria. In general, peptide dendrimeric structures manifest several advantages over corresponding peptides. In fact, peptide dendrimers are more easily synthesized, are able to preserve their active in low- and high-salt conditions, resist to proteases, are generally less cytotoxic, and act at lower concentrations.

A further aspect to be taken into consideration is the fact that dendrimers have shown the ability to inhibit biofilm formation. The antibiofilm activity has been proved against several bacteria such as *E. coli* or *P. aeruginosa* and with different dendrimeric molecules. Bacterial biofilms pose serious health problems as they are extremely difficult to eradicate since microbes embedded in the biofilm retain their pathogenicity by hiding from the host immune system defenses and also by increasing their resistance to bactericidal drugs. Bacterial biofilms formation is a frequent cause of hospital-acquired infection, often related to colonization of medical devices such as catheters. Antimicrobial dendrimers have been shown to be capable of preventing bacterial adhesion to surfaces, therefore, limiting subsequent biofilm development. Furthermore, the dendrimers may penetrate the exopolysaccharide matrix, deteriorate its integrity and destroy biofilm structure therefore acting also on mature biofilm and not only in preventing its formation [147,148,149]. In this direction, Bahar et al. showed, in dose dependent manner, that an arginine-tryptophan-arginine 2D-24 dendrimeric peptide was highly efficient in demolition of *P. aeruginosa* biofilms [150]. An additional group of antibacterial dendrimers is able to directly bind and neutralize bacterial toxins. Toxins released by specific gut bacteria represent the direct cause of several gastrointestinal diseases including cholera and traveler’s diarrhea. The bacterial toxins penetrate into target cells following specific binding to glycolipids present on the cell membrane. Inhibition of these proteins–carbohydrate interactions could prove to be advantageous to preclude the toxins from reaching their site of action, and thus avoid the consequential diarrhea. Thompson et al. developed an oligosaccharide-dendrimer, the oligo-GM1–PITC dendrimer that inhibited binding of cholera toxin B subunit and heat labile enterotoxin of *E. coli* to GM1-coated wells. In order to decrease the attachment capacity of the cholera toxin also galactose-functionalised dendrimers have been described [151,152]. A CBS dendrimer with three, four and six galabiose units, is highly active against shiga toxins produced by *E. coli* O157:H7 [153]. In vivo studies, using mice, have shown that dendritic compounds based on a glucose center linked with divalent sugar portions in each hydroxyl group are active against shiga toxins [154].

Besides their activity as antimicrobial compounds, dendrimers can be viewed as agents able to boost the curative potency of existing antibiotic drugs. For example, quinolones are not soluble in water, therefore are restricted to topical applications. PAMAM dendrimers have been used as drug carriers of quinolones (nadifloxacin and prulifloxacin) to increase the aqueous solubility. Results showed that both antibiotics retained their strong antimicrobial activities in the presence of dendrimers [155]. Dendrimers offer the opportunity to modify conventional drugs with the objective of boosting their efficacy. This can be easily obtained by loading drugs on dendrimers through physical adsorption, encapsulation in a polymer or matrix, or by chemical conjugation. The pharmacokinetics and antibacterial effect of the newly conceived drug can be greatly increased in comparison with the free drug [156]. An interesting study demonstrated the application of a multivalent strategy as a powerful way for reducing a ligand affinity limitation that can develop in vancomycin-resistant bacterial species. Fifth generation (G5) PAMAM dendrimers with attached vancomycin were analysed by surface plasmon resonance (SPR) to determine their binding avidity to two cell wall models, one representing a vancomycin-susceptible (d)-Ala-(d)-Ala and the other a vancomycin-resistant (d)-Ala-(d)-Lac cell wall precursor. These conjugates showed a noteworthy improvement in avidity in the cell wall models analysed, including the vancomycin-resistant model, where a five-fold increment in avidity could be observed in comparison to free vancomycin [157,158].

The variety of dendrimers available can allow the development of a large number of new antimicrobial products, with excellent characteristics of bioavailability and biodegradability. 

#### 4.2.2. Antiviral Activity

Viruses are obligate intracellular pathogens that supplant the host cell machinery to replicate, therefore, in order to avoid damage to vital cellular functions only some highly specific virus metabolic mechanisms offer the possibility to allow antiviral agents development. Furthermore, each virus possesses specific proteins and mechanisms, rendering the search for putative targets even more stringent. To improve the actual antiviral warfare, interdisciplinary research endeavours are imperative for the advancement of alternative strategies. 

Dendrimer applications offer an opportunity for an innovative delivery systems for antiviral administration, in particular for those drugs that act outside the cell membrane, which have as targets the early mechanisms of viral entry into susceptible cells [140]. In this scenario, the high molecular weight and multivalent binding efficiency of dendrimers allow the display of antiviral activity by interference between the virus-host cell interactions via steric shielding or competitive inhibition [141]. Since dendrimers are characterized by high surface-to-volume ratios, is it possible the combination and attachment of different antiviral compounds on the same structure. This strategy offers the chance of targeting multiple and precise biological sites, limiting the necessity of high doses of antiviral drugs and reducing side effects on healthy cells and tissues [141]. A more detailed description of dendrimers endowed with antiviral activity can be found in some recent reviews [159,160,161,162].

One of the most successful applications of dendrimers as antiviral agents is pictured by lysine dendrimers, of which SLP7013 is considered the most representative.

SLP7013 presents a core based on a divalent benzhydrylamineamide of l-lysine, which is encircled by four l-lysine layers (G4) terminated with 32 amine groups and further functionalized with naphthalene disulfonic acid groups. This dendrimer is commercialised as VivaGel^®^ by Starpharma [120,142] and has proved to possess a strong antiviral activity against herpes simplex viruses (both HSV-1 and HSV-2) and HIV. 

Considering that in vivo studies as vaginal microbicide showed that VivaGel is able to provide a partial protection lasting at least 12 h, while a complete protection can be reached for a period of more than 1 h, without any side effect, it seems to be an excellent alternative to conventional antivirals [147]. 

SPL7013 dendrimers also exhibit antiviral activity against HSV-2 strains resistant to penciclovir and acyclovir. Its functionalization with 32 different naphthyldisulfonic acid derivatives showed a dual action of this complex: inhibition of virus attachment to the host cell and block of viral replication with a drastic fall in HSV2 DNA synthesis in dendrimers-treated cells [161]. 

Other dendrimeric scaffolds have been studied against HSV, for example, peptide dendrimer SB105 and its derivative SB105_A10. Both are able to block infectivity of HSV-1 and HSV-2. These dendrimers are constructed starting from a tetrameric lysine hearth and 10-mer peptides attached on the exterior. The peptide dendrimers bind to heparan sulfates (HSV initial entry receptor) that are present on the surface of susceptible cells; therefore, the mechanism of action has been individuated in the block of the viral attachment which consequently prevents the whole infection process [141]. Interestingly, SB105_10A and acyclovir were shown to act in a synergic way, therefore opening the possibility of combined therapies. 

A further example of a peptide-derivatized dendrimer active against HSV exploits the antiviral activity of peptides directly derived by the viral surface glycoproteins. A poly(amide)-based dendrimer has been functionalized via the CuAAC using a modified gH625 (derived from HSV-1 glycoprotein H) peptide containing a PrA residue at the C-terminus (NH_2_-HGLASTLTRWAHYNA LIRAFX-CONH_2_). The peptidodendrimer carrying the gH625 peptide at its termini showed the average particle size to be 12 nm, and was confirmed by circular dichroism that the terminal peptides are still able to fold into an α-helix in a membrane-mimetic environment [148,149]. A multivalent display of gH625 on the dendrimer scaffold resulted in a six-fold increase of antiviral activity against HSV-1 and a two-fold increase against HSV-2 (the sequence identity between the two gH homologs is high, but not 100%) and a 100-fold increase in antiviral activity was recorded versus the unsupported peptide [150]. Due to the high level of complexity of the interaction between HSV envelope glycoproteins during the fusion process, it was not unequivocally identified the mechanism of antiviral action of the peptidodendrimer, which may act by directly binding to gH or may hinder the interactions of gH with other glycoproteins present on the virion envelope, such as gB or gD. Modification of a dendrimer scaffold with antiviral peptides constitutes a novel possibility where the intrinsic antiviral properties of a dendrimer can be coupled with known activity of antiviral peptides. In addition, peptide-dendrimers are amenable to provide multifunctionalized scaffolds to accommodate known therapeutic molecules to be directly delivered to target cells. Peptide dendrimers (SB105 and SB105_10A) have also been used against human cytomegalovirus (HCMV) and Human HPVs [151,152] and proved to be very efficient in inhibiting infectivity of both viruses and of different strains. Both viruses use heparan sulfate proteoglycans (HSPGs) as receptors to bind to target cells, therefore, peptides that block this attachment step may interfere with the ligand-receptor interactions. Competing with respiratory syncytial virus (RSV) for binding to cell surface may also explain the high activity of the same compound against this virus. Dendrimer SB105-A10 was the most potent inhibitor of RSV infectivity, with 50% inhibitory concentrations at less than 1 μM [153]. As described earlier, VivaGel is also active against HIV, but other dendrimers possess anti-HIV activity, such as fourth-generation PAMAM branched-naphthalene disulfonic surface groups decorating SPL2923 [147] or polysulfonated dendrimer BRI292 [154]. These dendrimers act on several biological mechanisms of HIV-1 infection; among them scientific evidences were reported for blocking the entry mechanism as well as later steps (reverse transcriptase/integrase) of the viral replicative cycle. 

Polyanionic CBS dendrimers (namely G3-S16 and G2-NF16) with sulphated and naphthylsulphonated end groups have been investigated in details for their anti-HIV potential [155,156,157,158]. It was demonstrated that these CBS dendrimers act on the inhibition of HIV-1 infection at the fusion and the entry levels by hampering the binding of viral particles to target cell surface and reducing membrane fusion. The target has been shown to be the gp120-CD4 interaction. G3-S16 and G2-NF16 are polyanionic compounds with disturb electrostatic interactions happening between HIV-1 envelope proteins, such as gp120, and their external functional groups with the result of perturbing the viral attachment step on target cells. CBS dendrimer have been successfully used in combination with other dendrimers (G2-STE16, G2-S24P and G2-S16) and other HIV antiviral drugs (tenofovir, TFV or maraviroc, MRV) for the application as topical microbicide against HIV. These combinations reached 100% HIV infectivity inhibition and showed a synergistic profile against different HIV-1 isolates [163].

These CBS dendrimers have also been very promising for their use in combination with other drugs [159,161] or for the addition of metal moieties in the dendritic scaffolds to increase the range of their applications due to the different properties of metals [163,164]. DC-SIGN (a C-type lectin receptor) mediated viral entry was exploited for inhibition of cellular uptake of viruses (HIV and Dengue). A group of glycodendrimers of different valency bearing different carbohydrates or glycomimetic DC-SIGN ligands was produced and tested for competition by Surface Plasmon Resonance, demonstrating bind efficiently within the micromolar range.

An interesting study tried to exploit the possibility of blocking viral encapsidation of HIV through the use of a novel “packaging inhibitor” based on a dendrimer-RNA nanocomplex. The mechanism of encapsidation, where HIV RNA is packaged into nascent virions, is driven by interactions between by the Gag nucleocapsid protein p7 and a region of the HIV genome known as the packaging signal (Ψ). A decoy RNA, mimicking the Ψ signal, complexed with a third-generation CBS dendrimer was shown to be efficiently delivered to lymphocytes and exerting a cytoprotective effect against HIV, nevertheless, with only a minor suppression of HIV viral load [164,165].

Rivero-Buceta et al. investigated HIV inhibition of infectivity mediated by pentaerythritol derived dendrimers with aminotriesters as branching units to obtain multiple (from 9 to 18) tryptophan on the periphery [144]. Glycoproteins gp120 and gp141 are supposed to interact with these dendrimers preventing viral entry [144]. The same dendrimers have been, successively, proven to be potent, specific, and selective inhibitors of the replication of the unrelated enterovirus A71, but their mechanism remains unclear [145]. The work and structure-activity relationship studies were extended to determine the basic traits that could be responsible for the important and dual (against HIV and enteroviruses) antiviral activity previously shown. In order to increase the antiviral action and analyse the mechanism of action, Martínez-Gualda et al. [146] prepared novel dendrimers functionalized with tryptamine and *N*-methyl Trp, showing that tyrosine dendrimers strongly inhibited EV7 replication compared to the *N*-methyl Trp dendrimers, while the latter were more active against HIV.

A further virus that has been analysed for inhibition studies using dendrimers is hepatitis C virus (HCV). Sepúlveda-Crespo et al. have synthesized a novel dendrimer, a second-generation CBS dendrimer characterized by a polyphenolic core and 24 sulfonate groups on the surface (G2-S24P). They have assessed that high dendrimer doses (2.5 μM) destabilize HCV virion architecture while low concentrations (50 nM) could inhibit early phases of HCV infection. The polyanionic nature of G2-S24P is important for its antiviral action, in fact, at low doses, mimicking the mechanism of action of the heparin, these dendrimers electrostatically bind the host cell glycosaminoglycans [166]. Concerning the fact that drugs resistance are recorded in the nucleosides analog stavudine for treatment in HIV, a potential application of dendrimers was achieved with the preparation PEGylated PAMAM G4 and G5 dendrimers complexed to stavudine. This preparation increase the release profile in the cells, enhancing efficacy with a reduction of drug side effects [167]. 

#### 4.2.3. Antiparasitic Activity 

Despite the huge literature among dendrimers applications, an overview of their activity again protozoan parasites is lacking. By definition parasites are organisms obligated to be in contact with hosts [168]. Parasitosis is one of the major causes of morbidity and mortality in developing countries, causing millions of deaths every year [169]. The spread of these diseases is mainly due to the slow development of new therapeutic strategies and the rapid diffusion of resistant species [170]. Moreover, due to the toxic effects mediated by the antiparasitic drugs, clinicians are obligated to prescribe low doses of them for prolonged therapeutic regimens [171], further increasing the emersion of chemotherapy resistant species. In this scenario, dendrimers could represent the ideal solution in terms of efficient drug stability and biodisponibility to be used in rural or remote regions, especially if a toxicity reduction compared to existing drugs is achieved. Peptide-dendrimers have been assayed for the treatment of leishmaniasis. Leishmania species are obligate intracellular protozoa that infect the antigen presenting cells (APC) which express MHC class II. Daftarian et al. have coupled a Pan-DR-binding epitope (PADRE) to a 5th generation PAMAM dendrimer generating a PADRE-Derivatized Dendrimers (PDDs) able to deliver amphotericin B (AmB) (currently the most effective antileishmanial drug) to the source of infection. PADRE is the CD4^+^ Th determinant that binds most of the murine and human MHC II molecules [172]. AmB, that is negatively charged, could be easily complexed with liposomal amphotericin B. Both in vitro and in vivo studies, PDD showed a 83% improvement in drug efficacy and a 10-fold increase in APC targeting along with a significant reduction of both parasite burden and toxicity [173]. This AmB dendrimeric nanocarrier could be considered an elegant model for reducing drug regimens for the treatment of leishmaniasis. A further example of the use of dendrimer technology to deliver AmB for treating leishmaniasis has been proposed by Jain et al. [174]. A mannosylated 5th generation PPI dendrimer has been constructed as carrier and for the proper delivery of AmB. Mannosylation is expected to help the delivery of AmB to macrophages through interaction of the dendrimer with mannose receptors highly scattered on the surfaces of mature macrophages. AmB, loaded into mannose-conjugated dendrimers, was proved to be more efficient in reducing the burden of the infection. The described dendrimeric compound may represent a valid alternative for targeted delivery of AmB in the treatment of severe infections as demonstrated by the enhanced cellular uptake by macrophage cells, the reduced toxicity and the significant improvement of the inhibitory activity against the parasites in both in vitro and in vivo studies [174]. Recently, anti-plasmodial activity of peptide-dendrimers has been evaluated. Falciparum malaria, caused by *Plasmodium falciparum*, represents one of the most widespread parasitosis worldwide and constitutes a serious public health problem especially for the rapid development of species resistant to the latest drugs discovered such as chlorocin and artemisinin [175]. Red blood cells, infected with plasmodial parasites, have an altered distribution of lipids in the plasma membrane. The infected cells evince a displacement of the anionic phosphatidylserine, negatively charged, from the inner leaflet to the outer leaflet of the bilayer. The anionic character renders the infected cells susceptible to antimicrobial peptides. Most of these peptides show low potency, stability and selectivity, therefore Kaushik et al. have improved the anti-plasmodium action of several peptides by dendrimerization, using dendritic structures of poly-lysine. The dendrimeric versions have shown high potency with IC50 values lower than peptides alone. These peptidodendrimers have also shown a selectivity greater than 35% with a good resistance index and low cytotoxicity [176].

## 5. Conclusions

In the last 20 years forward steps were achieved in the dendrimer field and their application. The last five years were fundamental for the deeper dendrimer conformational characterization and for their applications. Recent efforts were done in order to setup structure-controlled methodologies that will enable cost-effective, controlled assembly of nanostructures in a very routine manner. This approach should be paired with approaches that are able to produce a well define organic and inorganic nanostructures with dimensions ranging between 1 and 100 nm. In our vision, the final dendritic strategies should be the ability to produce nanoscale structures and devices with specific size, shape, and surface chemistry. The ability to tune easily their size, chemistry, topology and properties through chemical synthesis has led to their widespread use in a variety of technological applications. Furthermore, the possibility to design dendrimers with different properties and functions has prompted the medical community to look forward their exploitation for several biomedical applications. Although the application of dendrimers is still in its infancy compared to liposomes and other nanomaterials, they are already playing a key role with several therapeutic products based on dendrimer technology which are in clinical trials.
